# Clinical Characteristics and Outcomes of Patients Undergoing Transcatheter Tricuspid Valve Replacement Stratified by TRISCEND II Trial Eligibility

**DOI:** 10.1016/j.jscai.2026.104385

**Published:** 2026-03-24

**Authors:** Sahar Razmjou, Sureja Sundaralingam, Nathaniel Bowerman, Georgi Fram, John Dawdy, Tiberio Frisoli, James Lee, Pedro Arturo Villablanca, Brian O’Neill, Pedro Engel Gonzalez

**Affiliations:** Center for Structural Heart Disease, Henry Ford Hospital, Detroit, Michigan

**Keywords:** clinical outcomes, transcatheter tricuspid valve replacement, TRISCEND II eligibility

## Abstract

**Background:**

Clinical trials for transcatheter tricuspid valve replacement (TTVR) use strict eligibility criteria that often exclude real-world patients, who may have more advanced disease and different outcomes. We compared characteristics and short-term results between patients who would have been eligible versus noneligible for the TRISCEND II trial.

**Methods:**

We analyzed a single-center cohort of 112 patients undergoing TTVR for symptomatic tricuspid regurgitation (TR). Patients were stratified according to TRISCEND II inclusion and exclusion criteria. Baseline demographics and 30-day outcomes were compared between eligible (n = 47) and noneligible (n = 65) groups.

**Results:**

Eligible patients were older (83.2 years [IQR, 77.4-86.7] vs 76.3 years [IQR, 69.6-83.1]), while sex distribution and New York Heart Association (NYHA) class III/IV were similar (female: 68.1% vs 64.6%; NYHA III/IV: 76.6% vs 75.4%). TR severity was comparable. Noneligible patients had lower ejection fraction (56% vs 60%) and more prior heart failure hospitalizations (13.8% vs 4.3%). At 30 days, both groups showed significant TR reduction, but noneligible patients remained more symptomatic (NYHA III/IV: 31.0% vs 11.9%) and had lower Kansas City Cardiomyopathy Questionnaire scores (65.63 vs 70.83). All-cause mortality (8.2% vs 2.0%) and major bleeding (17% vs 12.3%) were numerically higher in the noneligible group, whereas stroke, pacemaker implantation, and reintervention rates were low and similar.

**Conclusions:**

Eligible patients had better cardiac function and were less symptomatic post-TTVR. Noneligible patients remained more symptomatic despite similar TR reduction, highlighting the need to consider real-world patient complexity when interpreting trial outcomes.

## Introduction

Tricuspid regurgitation (TR) is a common valvular disorder associated with progressive right heart failure, impaired functional capacity, and poor long-term outcomes.^1^^,^^2^ Until recently, treatment options for severe TR were limited, as isolated tricuspid valve surgery carries high operative risk and medical therapy often provides inadequate symptomatic relief.^3^ In this context, transcatheter tricuspid valve replacement (TTVR) has emerged as a promising therapeutic strategy, particularly for patients at high or prohibitive surgical risk.^4^, ^5^, ^6^, ^7^ On February 1, 2024, the EVOQUE valve system (Edwards Lifesciences) became the first and currently only FDA-approved TTVR device in the United States, based on results from the pivotal TRISCEND II randomized trial.^8^ This study demonstrated significant improvements in symptoms and quality of life with TTVR, although it also reported notable rates of adverse events, particularly major bleeding and new permanent pacemaker implantation.^5^

As with many randomized controlled trials, TRISCEND II applied strict inclusion and exclusion criteria, selecting a well-defined population that may not fully reflect the heterogeneity and complexity of patients treated in real-world clinical practice. The extent to which these trial results generalize to broader patient populations remains unclear, particularly among patients who would not have met trial eligibility criteria but still underwent TTVR based on clinical judgment. Although TTVR is rapidly evolving, real-world outcome data are limited, and predictors of symptomatic benefits and periprocedural complications remain poorly characterized in routine practice.

The objective of this study was to evaluate a contemporary, single-center cohort of patients undergoing TTVR and to stratify them based on their eligibility for the TRISCEND II trial. We sought to compare baseline clinical characteristics, echocardiographic profiles, and 30-day clinical outcomes between TRISCEND II eligible and noneligible patients.

## Methods

### Study cohort and procedural technique

This study retrospectively analyzed a single-center database of 112 patients who underwent TTVR for symptomatic TR at a single, tertiary care center between February 2024 and April 2025. Patient eligibility and treatment decisions were made by a multidisciplinary heart team, considering clinical characteristics, comorbidities, and procedural risk. All patients received optimized medical therapy, including diuretics and heart failure medications, prior to TTVR. Procedural details of TTVR were consistent with previously described techniques.^5^^,^^9^^,^^10^ Patients received oral anticoagulation for at least 6 months postoperatively.^9^^,^^10^ Clinical data were collected at baseline, discharge, and 30-day follow-up.

### TRISCEND II trial eligibility

Patients were stratified based on eligibility criteria from the TRISCEND II trial, which evaluated the safety and efficacy of TTVR as compared with medical therapy. Eligibility was adjudicated by review of preprocedural clinical data. Patients eligible for the TRISCEND II trial were required to meet all the following inclusion criteria: age ≥18 years; symptomatic functional or degenerative severe or greater TR despite optimized medical therapy (including stable oral diuretics); and deemed appropriate candidates for TTVR by a multidisciplinary heart team based on clinical and anatomic factors.

Exclusion criteria encompassed conditions that could compromise procedural success or patient safety, including severely reduced left ventricular ejection fraction (LVEF) (<25%), severe right ventricular dysfunction, prohibitive pulmonary hypertension, prior tricuspid valve surgery or intervention, presence of recent transtricuspid pacemaker leads with contraindications, significant concomitant valvular disease requiring intervention, active infection, hemodynamically significant pericardial effusion or intracardiac masses, severe renal or hepatic dysfunction, recent cardiovascular procedures, and other comorbidities limiting life expectancy or trial compliance. Additionally, anatomic factors preventing safe device delivery and deployment, inability to access the femoral venous route, or contraindications to study medications or device components were also exclusionary. The design of the TRISCEND II pivotal trial has been previously published.^11^ Patients were classified as TRISCEND II eligible or noneligible for subgroup analyses.

In this study, right ventricular systolic pressure (RVSP) was measured via echocardiography and used as a surrogate for pulmonary artery systolic pressure to evaluate pulmonary hypertension, aligning with current clinical practice.^12^

### Data collection and outcomes

Baseline demographics, comorbidities, echocardiographic, and procedural data were obtained through medical records. Clinical outcomes assessed at 30 days after tricuspid valve intervention included all-cause mortality, heart failure symptoms evaluated by the New York Heart Association (NYHA) functional classification, and functional status. Quality of life was measured using the Kansas City Cardiomyopathy Questionnaire (KCCQ) test. Major bleeding was defined according to the Mitral Valve Academic Research Consortium.^13^ Device-related events were any unfavorable medical occurrence linked to a medical device's use, design, or malfunction, suggesting it caused or could cause harm, injury, or death. Experienced echocardiographers performed all assessments in alignment with current professional society recommendations. The study protocol was approved by the local ethics committee, and all patient data were collected in accordance with institutional guidelines.

### Statistical analysis

Continuous variables were tested for normality using the Shapiro-Wilk test of normality and reported as mean ± SD or median (IQR) accordingly. Categorical variables were expressed as counts and percentages. Between-group comparisons for continuous variables used *t* tests or Kruskal-Wallis rank sum tests as appropriate; categorical variables were compared using Fisher exact tests. Changes from baseline to 30 days were assessed using paired tests (eg, Wilcoxon signed-rank test).

Statistical significance was defined as *P* <.05. Statistical analyses were conducted using RStudio version 2024.04.2.

## Results

**Figure 1:**
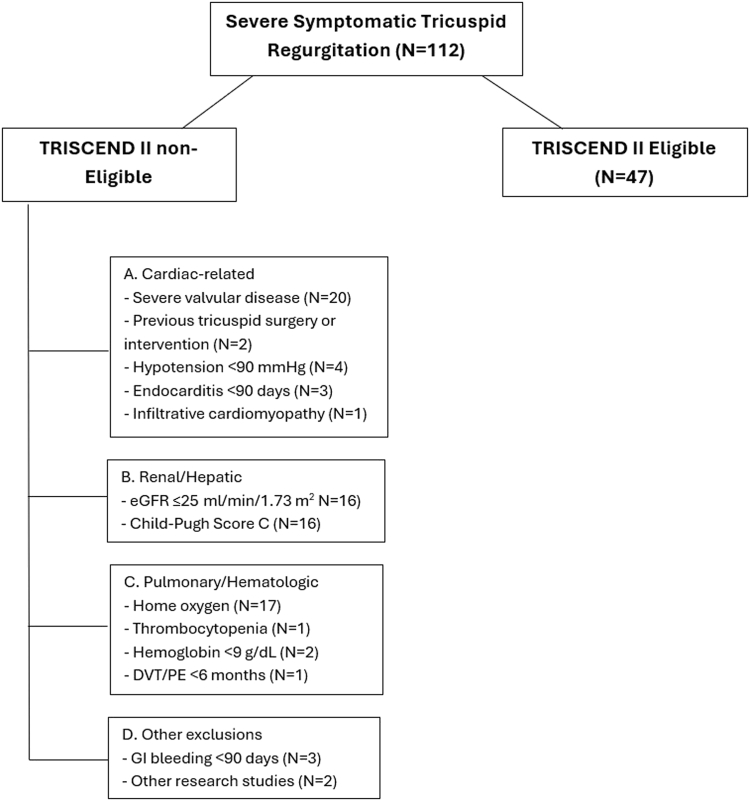
**Eligibility assessment for the TRISCEND II trial among patients with severe symptomatic tricuspid regurgitation (N = 112).** Flowchart demonstrating the distribution of patients assessed for eligibility in the TRISCEND II trial. Among 112 patients with severe symptomatic tricuspid regurgitation, 47 (42%) met the eligibility criteria, whereas 65 (58%) were considered noneligible because of various exclusion factors. All noneligible patients had at least 2 exclusion criteria (most had 2-4). eGFR, estimated glomerular filtration rate; mm Hg, millimeters of mercury; PE, pulmonary embolism; DVT, deep vein thrombosis; GI, gastrointestinal.

A total of 112 patients underwent TTVR between 2024 and 2025 at our institution. Among these, 47 patients (42%) met the eligibility criteria for the TRISCEND II trial, whereas 65 patients (58%) were considered noneligible (Figure 1). The reasons for ineligibility were multifactorial, with most patients meeting ≥2 exclusion criteria. The most common exclusions included significant cardiac comorbidities such as another concomitant severe valvular disease (30.7%) and previous tricuspid surgery (3.1%), advanced renal or hepatic dysfunction (eg, eGFR ≤25 mL/min/1.73 m^2^ or Child-Pugh class C; 24.6%), and pulmonary or hematologic factors, including the need for home oxygen (26.1%).

### Baseline characteristics

Baseline clinical and demographic characteristics are presented in Table 1. TRISCEND II eligible patients were significantly older than noneligible patients (83.2 years [IQR, 77.4-86.7] vs 76.3 years [IQR, 69.6-83.1]; *P* < .001). Noneligible patients had a significantly higher prevalence of end-stage renal disease (16.9% vs 0%, *P* < .01) and chronic obstructive pulmonary disease (44.6% vs 23.4%, *P* < .01). Although prior heart failure hospitalization in the past 12 months was more frequent among noneligible patients compared to eligible patients (13.8% vs 4.3%), this difference did not reach statistical significance (*P* = .11).

**Table 1:** Baseline patient characteristics.

Variable	TRISCEND eligible (n = 47)	TRISCEND noneligible (n = 65)	*P* value
Age, y	83.2 (77.4-86.7)	76.3 (69.6-83.1)	<.001
Female sex	32 (68.1)	42 (64.6)	.84
STS score, %	12.20 (18.13-18.40)	13.7 (8.34-22.21)	.40
Body mass index, kg/m^2^	26.6 (24.1-29.2)	28.2 (24.0-33.0)	.22
NYHA functional class III or IV	36 (76.6)	49 (75.4)	>.99
KCCQ score	48.3 ± 23.49	44.5 ± 24.18	.41
Prior heart failure hospitalization in the last 12 months	2 (4.3)	9 (13.8)	.12
BNP, pg/mL	344 (250-510)	515 (212-1126)	.08
Tricuspid regurgitation grade			.002
None/trace	0 (0.0)	0 (0.0)	
Mild	0 (0.0)	0 (0.0)	
Moderate	0 (0.0)	11 (16.9)	
Severe	47 (100.0)	54 (83.1)	
LVEF, %	60.0 (55.0-63.0)	56.0 (40.8-61.5)	.016
Atrial fibrillation	36 (76.6)	51 (78.5)	.82
Pacemaker	13 (27.7)	20 (30.8)	.83
End-stage renal disease	0 (0.0)	11 (16.9)	.003
Chronic obstructive pulmonary disease	11 (23.4)	29 (44.6)	.028
Right ventricular systolic pressure	34.6 (± 10.96)	41.9 (± 12.72)	.003
Medications			
Diuretics	40 (85.1)	51 (78.5)	.47
Spironolactone	8 (17.0)	17 (26.2)	.36
Beta blocker	35 (74.5)	50 (76.9)	.83
SGLT2 inhibitor	6 (12.8)	18 (27.7)	.07
ACE inhibitors	10 (21.3)	2 (3.1)	.004
Oral anticoagulant	38 (80.9)	51 (78.5)	.82

Values are n (%), mean ± SD, or median (IQR). GDMT data presented in Table 1 reflect pre-TTVR baseline therapy. Postprocedural GDMT adjustments were not available across patients and therefore were not analyzed.

ACE, angiotensin-converting enzyme; BNP, B-type natriuretic peptide; KCCQ, Kansas City Cardiomyopathy Questionnaire; LVEF, left ventricular ejection fraction; NYHA, New York Heart Association; SGLT2, sodium-glucose cotransporter-2; STS, Society of Thoracic Surgeons.

The surgical risk, as assessed by the Society of Thoracic Surgeons score, was numerically lower in the eligible group (median 12.20% vs 13.7%). Although both groups comprised predominantly women and had similar NYHA class III/IV burden at baseline (∼75%), eligible patients had significantly higher LVEF (60% [IQR, 55.0%-63.0%] vs 56% [IQR, 40.8%-61.5%]; *P* < .05]) and significantly lower RVSP (34.4 ± 10.96 vs 41.9 ± 12.72 mm Hg, *P* < .01). The baseline KCCQ score was slightly higher in the eligible cohort (48.96 [IQR, 34.38-63.54] vs 44.01 [IQR, 26.95-61.07]; *P* = .41), although not statistically significant. Baseline TR severity was high in both groups, with over 80% having severe or greater TR.

### Procedural success and clinical outcome

**Figure 2:**
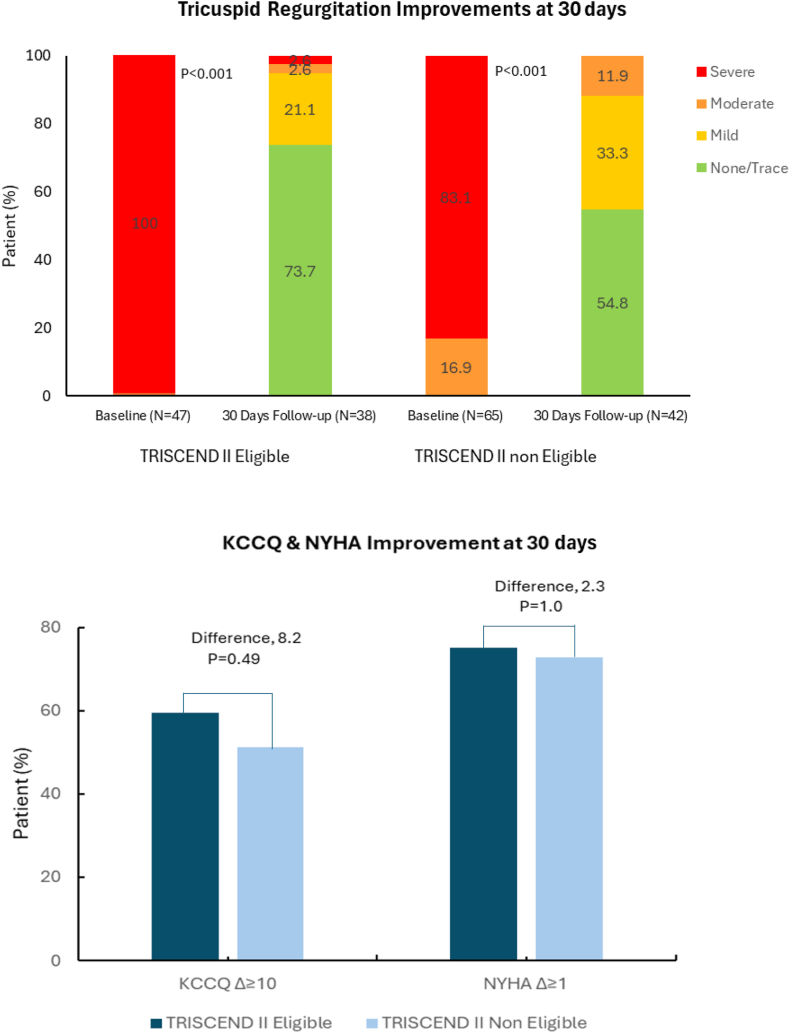
**Tricuspid regurgitation severity and clinical improvement at 30 days in TRISCEND II eligible vs noneligible patients.***P* value is based on the Wilcoxon signed-rank test. KCCQ, Kansas City Cardiomyopathy Questionnaire; NYHA, New York Heart Association.

Procedural success was high in both groups, with effective TR reduction achieved in all patients. At 30-day follow-up, a residual TR grade of ≤mild was observed in 94.8% of TRISCEND-eligible patients and 88.1% of noneligible patients (Figure 2). None/trace TR was achieved in 73.7% of eligible patients and 54.8% of noneligible patients, whereas moderate or greater TR persisted in only 5.2% and 11.9%, respectively. Device malposition, migration, embolization, or related complications were low in both groups.

Clinical outcomes at 30 days are shown in Table 2. There were no myocardial infarctions reported in either group. Stroke and reintervention were rare, occurring in only 1 patient each from the TRISCEND noneligible group. Rates of major bleeding and new pacemaker implantation were comparable between groups. Overall, all-cause mortality was numerically higher in the noneligible group (8.2% vs 2.0%, *P* = .21), though this difference was not statistically significant. The deaths that occurred were mostly cardiac- or procedure-related. One patient died at the time of the procedure because of entrapment of the delivery system, resulting in hemorrhagic pericardial effusion and shock. One patient died within 1 week because of bleeding, and 2 patients experienced cardiac arrest within 1 to 2 weeks following the procedure. Two additional deaths occurred approximately 1 month after the procedure: 1 from an unknown cause following an initially uncomplicated course, and 1 because of noncardiac causes.

**Table 2:** Clinical outcomes at 30-day follow-up.

Variable	TRISCEND eligible (n = 47)	TRISCEND noneligible (n = 65)	*P* value
All-cause mortality	1 (2.0)	5 (8.2)	.40
Reintervention	0 (0.0)	1 (1.6)	>.99
NYHA functional class III or IV	5 (11.9)	13 (31.0)	.07
TR grade			.11^a^
None/trace	28 (73.7)	23 (54.8)	
Mild	8 (21.1)	14 (33.3)	
Moderate	1 (2.6)	5 (11.9)	
Severe	1 (2.6)	0 (0.0)	
Lost to follow-up	9	23	
TR grade reduction (residual TR)	38 (100)	42 (100)	>.99
KCCQ score	65.3 ± 23.14	60.2 ± 23.94	.35
Bleeding - major	8 (17.0)	8 (12.3)	.59
Pacemaker	8 (17.0)	9 (13.8)	.79
Stroke	0 (0.0)	1 (1.5)	>.99
Myocardial infraction	0 (0.0)	0 (0.0)	>.99
Device malposition/migration/embolization	0 (0.0)	1 (1.5)	>.99

Values are N (%), mean ± SD, or median (IQR).

KCCQ, Kansas City Cardiomyopathy Questionnaire; NYHA, New York Heart Association; TR, tricuspid regurgitation.

*P* values were determined from 1-way ANOVA, Kruskal-Wallis test, or Fischer exact test, as appropriate.

a *P* value excludes the lost to follow-up.

Functional improvement was notable in both groups. The proportion of patients in NYHA class III or IV decreased from 76.6% and 75.4% at baseline to 11.9% in eligible and 31.0% in noneligible patients. Although not statistically significant, the KCCQ score improved more in the eligible group (70.83 [IQR, 48.96-85.42] vs 65.63 [IQR, 42.97-77.87]). As shown in Figure 2, a greater proportion of TRISCEND II eligible patients demonstrated improvements in both KCCQ (≥10-point increase: 59.5% vs 51.3%%, respectively) and NYHA class (≥1-class improvement: 75% vs 72.7%, respectively), though these differences also did not reach statistical significance.

### Adverse events

**Figure 3:**
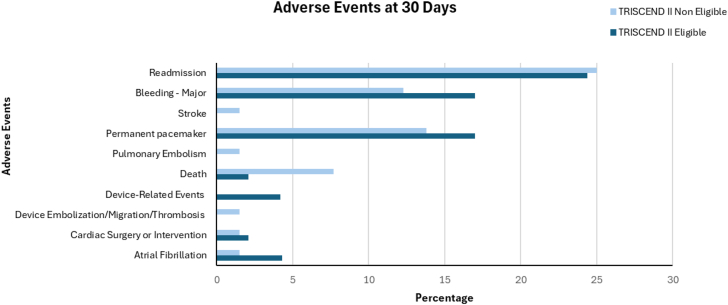
**Adverse events in TRISCEND II eligible and noneligible patients.**

Adverse events at 30 days are summarized in Figure 3. Major bleeding occurred in 17% of eligible patients and 12.3% of noneligible patients (*P* = .58). The incidence of permanent pacemaker implantation was comparable between groups, observed in 17% of eligible patients and 13.8% of noneligible patients (*P* = .79).

Device-related complications, including embolization, migration, or thrombosis, were not reported in the eligible cohort but occurred in 1.5% of noneligible patients. The overall incidence of device-related events was low, occurring in 4.2% of eligible patients and in none of the noneligible patients.

Atrial fibrillation was reported more frequently in eligible patients (4.3% vs 1.5%). Stroke occurred only in the noneligible cohort (0.0% vs 1.5%), as did pulmonary embolism (0.0% vs 1.5%). The need for cardiac surgery or reintervention was low and similar in both groups (2.1% vs 1.5%). All-cause mortality at 30 days was 2.0% in the eligible group compared to 8.2% in the noneligible group (*P* = .39). Readmission rates were 24.4% of eligible patients and 25% of noneligible patients.

In summary, the overall incidence of adverse events was low across both cohorts. However, numerically higher rates of mortality and certain complications were observed in the noneligible group.

### Major adverse cardiac events

The incidence of 3-point major adverse cardiac events (MACE), defined as cardiovascular death, nonfatal myocardial infarction, or nonfatal stroke, was 0% in patients eligible for the TRISCEND II trial and 4.9% in noneligible patients at 30 days post-TTVR. This included 2 cardiovascular deaths and 1 nonfatal stroke in the noneligible cohort; no myocardial infarctions were reported in either group. This difference may reflect the exclusion of higher-risk individuals from trial eligibility criteria. Nonetheless, the overall incidence of MACE remained low across both cohorts, reinforcing the short-term safety of TTVR when applied to appropriately selected patients.

## Discussion

The EVOQUE system, the first TTVR device approved in the US, has shown significant benefits in reducing TR, improving symptoms, and promoting right ventricular remodeling. However, it is also associated with notable complication rates, including severe bleeding and pacemaker implantation.^5^ Despite rapid advancements and implementation, real-world data on outcomes and predictors of complications in trial eligible vs nontrial eligible patient remain limited. Our study provides a comprehensive assessment of real-world outcomes following TTVR using the EVOQUE system, stratified by eligibility for the TRISCEND II pivotal trial. Our findings underscore key differences in baseline clinical profiles, procedural outcomes, and short-term safety between patients who met trial inclusion criteria and those who did not. Importantly, both groups experienced significant symptomatic and functional improvements at 30 days (Central Illustration).

**Central Illustration:**
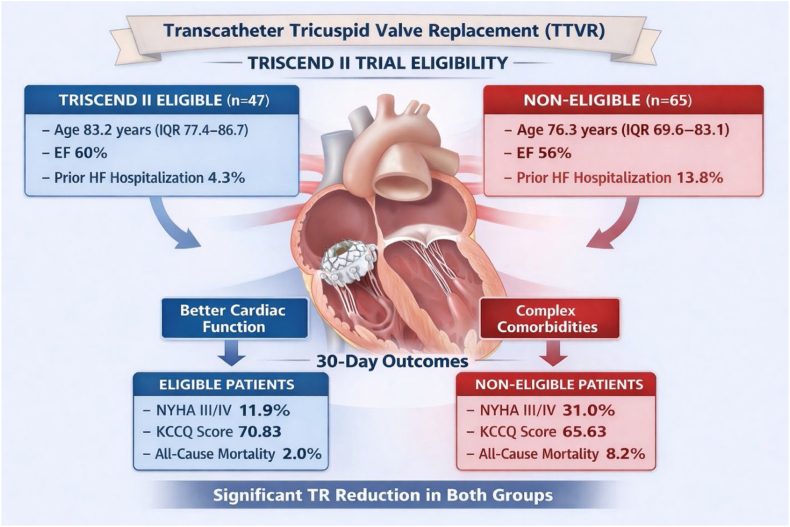
**Baseline characteristics and30-dayoutcomes after transcatheter tricuspid valve replacement (TTVR) with the EVOQUE system according to TRISCEND II trial eligibility.** Both eligible and noneligible patients achieved significant tricuspid regurgitation (TR) reduction, while noneligible patients remained more symptomatic at 30 days. EF, ejection fraction; HF, heart failure; KCCQ, Kansas City Cardiomyopathy Questionnaire; NYHA, New York Heart Association.

Approximately 58% of our TTVR population would have been excluded from participation in TRISCEND II because of comorbidities or anatomic contraindications, highlighting a substantial proportion of real-world patients who fall outside traditional trial frameworks. These individuals were younger on average but had a significantly higher burden of comorbid conditions, including advanced renal disease (16.9% vs 0%, *P* < .05) and chronic pulmonary disease (44.6% vs 23.4%, *P* < .05). They also exhibited markers of more advanced cardiopulmonary compromise, such as elevated RVSP and lower LVEF. These findings are consistent with prior observations that randomized controlled trials often enroll a more selected, less comorbid population than that encountered in routine clinical practice.^14^, ^15^, ^16^, ^17^ In fact, recent data from Abushouk et al^15^ underscore that only 10.7% of patients referred for transcatheter tricuspid therapy at a high-volume center were ultimately eligible for enrollment in trials like TRISCEND, TRILUMINATE, or CLASP-TR. This low eligibility rate was largely due to comorbid conditions such as chronic kidney disease, pacemaker leads, severe pulmonary hypertension, or unfavorable anatomy, reflecting the exclusion criteria that defined our own noneligible cohort. These insights further highlight the continued need for postapproval registries to continue to support the use of these technologies in a broader population of patients.

Despite these differences, procedural success was high and consistent across both groups, with effective TR reduction achieved in over 95% of patients. This supports previous studies demonstrating that the EVOQUE system can achieve effective TR reduction even in anatomically and clinically complex patients.^5^^,^^6^^,^^18^ The degree of residual TR at 30 days, including rates of trivial or no regurgitation, was comparable between eligible and noneligible patients, demonstrating the feasibility of TTVR even in anatomically or clinically complex cases. This aligns with outcomes from real-world transcatheter edge-to-edge repair (TEER) registries, such as the BRIGHT study, which have demonstrated similarly durable TR reduction.^17^ Consistent findings have also been reported in a recent multicenter European study of the EVOQUE TTVR system, conducted in a real-world setting, where 98.4% of patients achieved TR reduction from severe or greater to mild or none at 30 days in 176 patients.^19^

Clinically, both groups demonstrated marked improvement in functional capacity and quality of life. The majority of patients experienced at least 1 NYHA class improvement, with eligible patients showing a slightly higher proportion of patients reaching class I or II status (88.1% vs 69%). Although these trends suggest a benefit in the eligible group, it is important to note that the KCCQ confidence intervals were relatively wide, likely because of high variability in individual KCCQ responses, regardless of eligibility status. These findings are consistent with TRISCEND II, which reported significant improvements in both functional status and health-related quality of life following TTVR.^2^ Similar improvements were observed in the European multicenter study of the EVOQUE TTVR system, where the proportion of patients in NYHA class I or II increased from 20.2% at baseline to 79.7% at 30-day follow-up, further reinforcing the symptomatic benefit of TTVR across a broad spectrum of patients.^19^ Our data extend these results by confirming similar benefits in a broader, more medically complex population.

Bleeding complications occurred in both TRISCEND-eligible and -noneligible patients, with major bleeding reported in 17% and 12.3% of patients, respectively (*P* = .58). These rates are higher than those previously reported in both clinical trials and real-world studies of TTVR. For example, the TRISCEND II study reported a 10.4% rate of major bleeding at 30 days, and a recent multicenter European analysis of commercial EVOQUE use reported 9.7%.^5^^,^^18^^,^^19^ The incidence of major bleeding in our cohort, despite a higher comorbidity burden in the noneligible group, may reflect ongoing improvements in procedural technique, anticoagulation management, and patient selection across both groups. These findings underscore the need for continued optimization of bleeding risk management in all patients undergoing TTVR.

In our cohort, device-related events were rare, occurring in 4.2% of TRISCEND-eligible patients. Device migration occurred in 1.5% of the noneligible group, suggesting that anatomic and hemodynamic challenges in this subgroup may be associated with a slightly increased technical risk. No other device malposition, embolization, or thrombosis events were observed in either cohort.

Although permanent pacemaker implantation occurred at similar rates between groups (17% vs 13.8%), other complications and adverse events, such as death, were slightly higher among noneligible patients. In comparison, the TRISCEND trial reported a 30-day new permanent pacemaker implantation rate of 24.7%, whereas real-world data from a European multicenter study of commercial EVOQUE use showed a rate of 18.9% among pacemaker-naïve patients.^5^^,^^19^ These findings highlight that although pacemaker implantation rates in our cohort were somewhat lower, noneligible patients still exhibited greater procedural vulnerability overall, underscoring the importance of comprehensive risk assessment in real-world TTVR candidates.

The incidence of 30-day MACE remained low overall, with events occurring only in the noneligible group (4.9% vs 0.0%). This difference likely reflects the exclusion of patients with greater comorbid burden, reduced organ function, or complex anatomy from trial eligibility, factors that can increase periprocedural risk. Nonetheless, the relatively low event rate in both groups suggests that TTVR can be performed safely in appropriately selected patients outside of traditional trial criteria, provided that procedural planning and postoperative care are optimized.

Our findings have important implications for both clinical practice and future research. The data here inform the real-world applicability of TTVR in selected patients beyond those included in randomized trials. These insights also highlight opportunities to refine future trial designs by incorporating broader inclusion criteria, adaptive risk models, and patient-centered end points. Moreover, real-world registries and postapproval studies will be critical in capturing longer-term outcomes, particularly among high-risk subgroups that remain underrepresented in current evidence bases.

### Limitations

This study has several limitations that merit consideration. The sample size, particularly when stratified by eligibility status, reduces the statistical power to detect certain differences, especially in adverse events, because the incidence was overall low. Additionally, follow-up was limited to 30 days, preventing assessment of long-term outcomes and the durability of observed benefits. Other important limitations include the absence of a core laboratory for standardized assessment of TR severity and other imaging metrics, which may introduce variability. Potential confounders related to eligible versus noneligible status may not have been fully captured, reflecting the real-world complexity of managing patients with severe TR. Furthermore, clinical events occurring outside our health system could have been missed despite thorough efforts to adjudicate all known events.

Despite these limitations, this study offers valuable real-world insights and generates hypotheses for future research in this challenging patient population.

## Conclusion

In conclusion, patients meeting TRISCEND II eligibility criteria undergoing TTVR demonstrate better baseline cardiac function, fewer comorbidities, and superior short-term clinical outcomes compared to noneligible patients. Nevertheless, the high procedural success and meaningful symptom improvement observed in noneligible patients, as well as the relatively low rate of adverse events, provide important data to inform the use of TTVR in a broader patient population. These findings underscore the need for ongoing evaluation of real-world populations to inform patient selection, procedural planning, and trial design.

## Declaration of competing interest

The authors declared no potential conflicts of interest with respect to the research, authorship, and/or publication of this article.
